# Building a model for predicting metabolic syndrome using artificial intelligence based on an investigation of whole-genome sequencing

**DOI:** 10.1186/s12967-022-03379-7

**Published:** 2022-04-28

**Authors:** Nai-Wei Hsu, Kai-Chen Chou, Yu-Ting Tina Wang, Chung-Lieh Hung, Chien-Feng Kuo, Shin-Yi Tsai

**Affiliations:** 1 Department of Medicine, Mackay Medical College, New Taipei City, Taiwan; 2 Department of Laboratory Medicine, MacKay Memorial Hospital, Taipei City, Taiwan; 3 Department of Nursing, MacKay Junior College of Medicine, Nursing and Management, New Taipei City, Taiwan; 4 Division of Infectious Diseases, Department of Internal Medicine, Mackay Memorial Hospital, Taipei, Taiwan; 5 Department of Health Policy and Management, Johns Hopkins Bloomberg School of Public Health, Johns Hopkins University, Baltimore, Maryland 21205 USA; 6 Institute of Biomedical Sciences, Mackay Medical College, New Taipei City, Taiwan; 7 Institute of Long-Term Care, Mackay Medical College, New Taipei City, Taiwan

**Keywords:** Circadian rhythm, Metabolic syndrome, Whole-genome sequencing, Deep learning

## Abstract

**Background:**

The circadian system is responsible for regulating various physiological activities and behaviors and has been gaining recognition. The circadian rhythm is adjusted in a 24-h cycle and has transcriptional–translational feedback loops. When the circadian rhythm is interrupted, affecting the expression of circadian genes, the phenotypes of diseases could amplify. For example, the importance of maintaining the internal temporal homeostasis conferred by the circadian system is revealed as mutations in genes coding for core components of the clock result in diseases. This study will investigate the association between circadian genes and metabolic syndromes in a Taiwanese population.

**Methods:**

We performed analysis using whole-genome sequencing, read vcf files and set target circadian genes to determine if there were variants on target genes. In this study, we have investigated genetic contribution of circadian-related diseases using population-based next generation whole genome sequencing. We also used significant SNPs to create a metabolic syndrome prediction model. Logistic regression, random forest, adaboost, and neural network were used to predict metabolic syndrome. In addition, we used random forest model variables importance matrix to select 40 more significant SNPs, which were subsequently incorporated to create new prediction models and to compare with previous models. The data was then utilized for training set and testing set using five-fold cross validation. Each model was evaluated with the following criteria: area under the receiver operating characteristics curve (AUC), precision, F1 score, and average precision (the area under the precision recall curve).

**Results:**

After searching significant variants, we used Chi-Square tests to find some variants. We found 186 significant SNPs, and four predicting models which used 186 SNPs (logistic regression, random forest, adaboost and neural network), AUC were 0.68, 0.8, 0.82, 0.81 respectively. The F1 scores were 0.412, 0.078, 0.295, 0.552, respectively. The other three models which used the 40 SNPs (logistic regression, adaboost and neural network), AUC were 0.82, 0.81, 0.81 respectively. The F1 scores were 0.584, 0.395, 0.574, respectively.

**Conclusions:**

Circadian gene defect may also contribute to metabolic syndrome. Our study found several related genes and building a simple model to predict metabolic syndrome.

**Supplementary Information:**

The online version contains supplementary material available at 10.1186/s12967-022-03379-7.

## Background

Metabolic syndrome (MetS) is a cluster of commonly concurrent metabolic risk factors associated with cardiovascular disease and type 2 diabetes mellitus, including: elevated blood pressure, atherogenic dyslipidemia, insulin resistance, and central obesity (measured as waist circumference with ethnic specific values). Thus, metabolic syndrome can eventually lead to conditions such as Chronic Kidney Disease (CKD) and atherosclerotic cardiovascular disease [1].

Risk factors of metabolic syndrome include family history, smoking, obesity, lack of physical activity and lifestyle factors [2, 3]. Sugar-sweetened soft drinks have been reported to increase risk [4, 5]. Children who have an increased body mass index (BMI), systolic blood pressure (SBP) and triglyceride levels are believed to be at higher risk of developing MetS in middle age [6].

The prevalence of metabolic syndrome is highest among those who are overweight and obese. The International Diabetes Federation (IDF) estimated that one-quarter of the world’s population suffers from metabolic syndrome. Taking age into consideration, metabolic syndrome appears to be most common in the elderly in those who are over 60 of age [2]. On average, the prevalence of metabolic syndrome in adults is about 23% [7]. A national survey done in Taiwan, the Nutrition and Health Survey in Taiwan (NAHSIT) 2005–2008 showed a significant increase in the prevalence of MetS from 13.6% (1993–1996) to 25.5% (2005–2008) for males, and 26.4% to 31.5% in females respectively over a period of 10–15 years. The relationship between diabetes, high blood pressure, heart disease, cerebrovascular disease and metabolic syndrome is inseparable, as these conditions and or their associations are among the top ten causes of death in Taiwan [8].

Circadian rhythm plays an important role in endocrine secretion, body temperature [9]. An important aspect of circadian rhythms is that they persist in the absence of external cues [10]. Circadian genes which express periodically in an approximate 24- hour period help to regulate the genes of metabolism [11–13]. Previous animal models have showed that knockout of specific circadian gene will influence the circadian behavior. The recognition that multiple transcription factors function in the circadian gene, and that each of these has thousands of genomic DNA binding sites. Each of the circadian genes contributes directly to individual gene regulation in addition to its role in the reciprocal and homeostatic regulation of other clock genes by transcriptional-translational feedback loops that define the clock itself [14]. Many disease have been found to related to circadian genes including Alzheimer’s diseases, Parkinson disease [15], atherosclerotic disease [16] or viral infection.

Circadian rhythm also affects oxidative stress, too. If the human body or cells experience significant stress, their ability to regulate internal systems, including redox levels and circadian rhythms, may become impaired [17]. Animal studies have showed that risperidone may reset circadian rhythm [18]. Risperidone was found to induce cytotoxicity via rising reactive oxygen species (ROS), mitochondrial potential collapse, lysosomal membrane leakiness, GSH depletion and lipid peroxidation, and some antioxidant like coenzyme Q10 or N-acetyl cysteine may have a role as a therapeutic options [19]. Circadian rhythm also has played a role in liver lipid metabolism and renin angiotensin system [20] and chronic fatigue syndrome [21, 22]. The timing of statins therapy may influence the effect [23]. Renin angiotensin system was found to induce oxidative stress and fibrogenic cytokine [24]. Altering circadian rhythm may have a huge amount of influence over treatment of chronic liver diseases.

Increasing evidence shows that circadian clock genes may contribute to the development of metabolic syndrome [25, 26]. Circadian clocks regulate the timing of biological events including the sleep–wake cycle, energy metabolism, and secretion of hormones, etc. In an association and interaction analysis from Lin et al., the study proposed that many of these core circadian clock genes impacts metabolic activity and metabolism, which may lead to metabolic syndrome [27]. We targeted the core circadian clock genes that have been potentially linked with MetS.

## Method

### Study population

We used Taiwan Biobank (TWB) NGS cohort as our study population. TWB collects lifestyle, genomic data, and represent diseases from Taiwan residents. TWB recruits community-based volunteers who are 30 to 70 years of age and have no history of cancer. This cohort was based on the recruitment and monitoring from the general Taiwanese population, and has been utilized in previous genetic studies [28]. Our study included 642 TWB individuals who have whole genome sequence (WGS) data.

### Metabolic syndrome definition

According to the new International Diabetes Federation (IDF) definition, metabolic syndrome must meet the criteria of having central obesity (measured in waist circumference specific to the ethnic values, see below) plus 2 of the following 4 factors:

- Triglycerides ≥ 150 mg/dL (1.7 mmol/L) or taking drug treatment for elevated triglycerides
- Fasting glucose ≥ 100 mg//dL or previously diagnosed Type 2 Diabetes Mellitus
- Reduced high-density lipoprotein (HDL) cholesterol or drug treatment for reduced HDL cholesterol:
- in men, < 40 mg/dL (1.0 mmol/L)
- in women, < 50 mg/dL (1.3 mmol/L)

Elevated blood pressure demonstrated by any of the following:

- systolic blood pressure ≥ 130 mm Hg or
- diastolic blood pressure ≥ 85 mm Hg or
- antihypertensive drug treatment in a patient with a history of hypertension.

As our study took place in Taiwan and our data from the Taiwan Biobank, we used the ethnic specific values for waist circumference according to the “South Asians” and “Chinese” groups, where central obesity was defined as having a waist circumference of ≥ 90 cm in males and ≥ 80 cm in females.

### Finding suspected single nucleotide polymorphisms

This analysis analyzed a total of 642 cases of WGS with the illumina platform (of which 123 were defined as metabolic syndrome patients) with target genes: ALAS1, APOA5, ARNTL, BUD13, CETP, CLOCK, CRY1, CRY2, CSNK1D, CSNK1E, GSK3B, LIPA, NPAS2, NR1D1, PER1, PER2, PER3, RORA, RORB, RORC, SMAD2, SMAD3, SMAD4, TGFB2, TGFB3, TGFBR2 and other genes within the range of SNPs for analysis. The range of SNP was set between 17 and 37 (average of > 30) with Qual >  = 30 [29].

However, during this experiment, the range of data analysis was larger than originally expected due to a problem of the single nucleotide polymorphism (SNP) range set for CSNK1E. The definition of metabolic syndrome was primarily based on the physiological data of Taiwan's BioBank database. After it was imported into the SQL server, the patients were grouped with the database language as the basis for subsequent analysis.

The frequency of occurrence of single-strand, double-strand variation or non-variation in each group was counted. Subsequently the mathematical formula was written in Python and statistical analysis was applied to calculate the 95% confidence interval and the chi-square or Fisher’s Exact test to calculate the p value. After identifying significant SNPs, we conducted subgroup analysis to find out whether these SNPs are related to hypertension, low HDL level, diabetes or high TG level. Bonferroni Correction was used to tackle Multiple hypothesis testing, due to there are 5 category of metabolic syndrome, alpha value was set to 0.5/5 = 0.1.

### Statistical analyses

P values for continuous variables were calculated using student’s t test. Categorical variables were compared using the chi-square test or exact test. Given the exploratory nature of this study, P < 0.05 was considered statistically significant. We use caret package in R software version 4.04 for model prediction. We also use C#, python and MySQL for data manipulation.

### Creation of genome-based prediction model

**Fig. 1:**
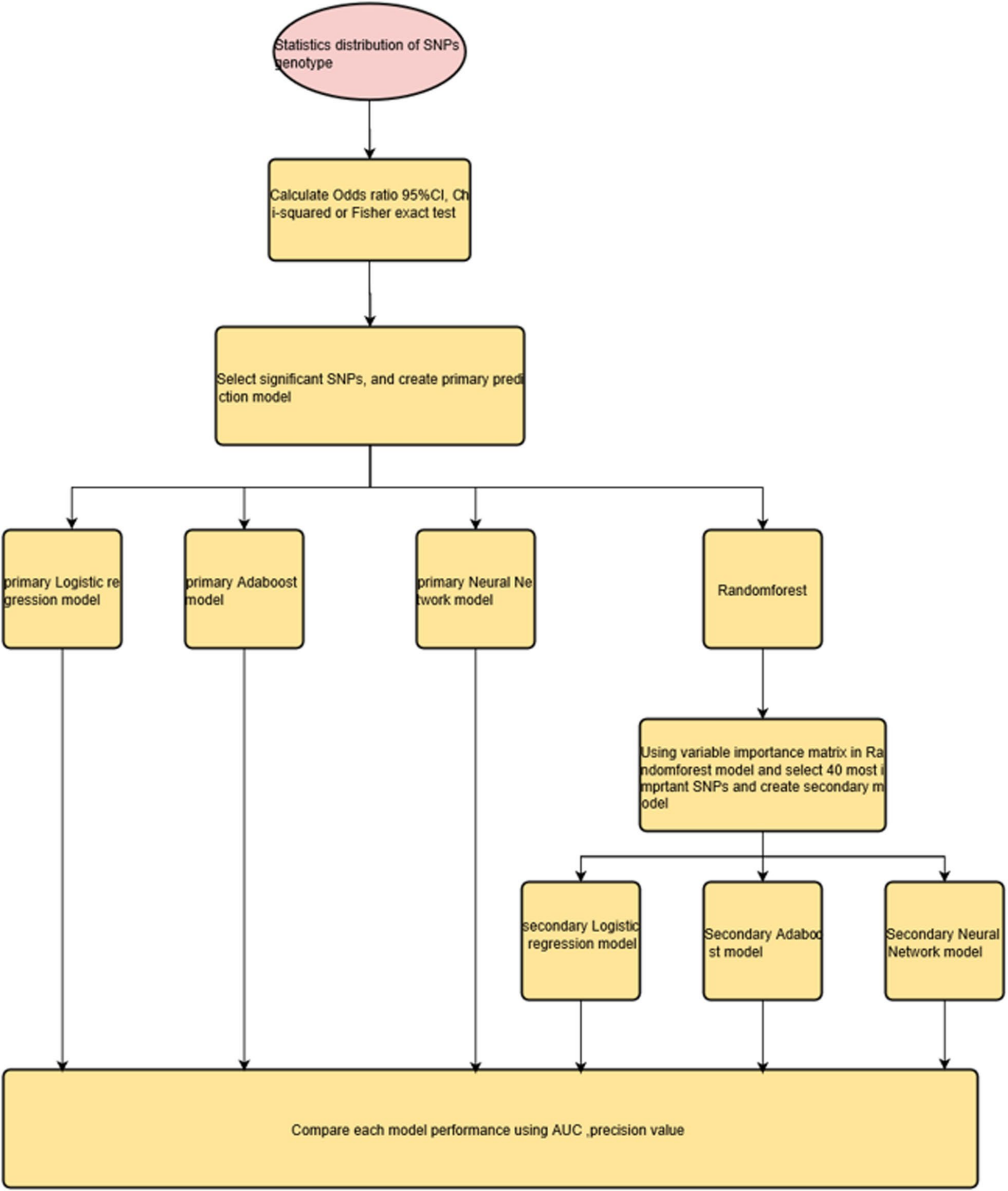
Flow diagram for model building

We use significant SNPs to create a metabolic syndrome prediction model. Logistic regression, random forest, adaboost, and neural network were used to predict metabolic syndrome. The data was used for training set and testing set using five-fold cross validation. We assumed that there was a cumulative effect on SNPs, so we take homozygous equal to 2, heterozygous equal to 1 and wild type as 0. Since weight may be influenced by these genes, weights are not use as a covariate [30]. Besides the four models mentioned above, we selected 40 importance SNPs according to random forest important matrix, then using them to create another three model using the logistic regression, adaboost and neural network method (Fig. 1). We used a simple neural network with one layer and size 10 units in the hidden layer and decay equals to 0. Each model was evaluated with the following criteria: area under the receiver operating characteristics curve (AUC), precision, F1 score, and average precision (the area under the precision recall curve).

## Results

### Baseline characteristic of metabolic syndrome individuals and control group

Among 642 study population, there were 124 individuals with metabolic syndrome and 518 individuals without metabolic syndrome. The mean age of metabolic syndrome cohort was 51 years old, and the mean age of non-metabolic syndrome cohort was 44 years old. We have found that the values of waistline, blood pressure, triglyceride level, hemoglobin A1C, fasting glucose and diabetes mellitus percentage in metabolic syndrome patient is higher than those without metabolic syndrome. In addition, the high-density lipoprotein value in metabolic syndrome is lower than those without metabolic syndrome which is corresponding to metabolic syndrome definition (Table 1).

**Table 1:** Baseline characteristic of the patients

	No metabolic syndrome (N = 518)	Metabolic syndrome (N = 124)	P-value
AGE(Years)	44.48 ± 10.19	51.76 ± 10.02	< 0.001
HEIGHT(cm)	165.44 ± 7.89	165.26 ± 8.63	0.831
WEIGHT(Kg)	64.7 ± 11.44	75.92 ± 12.89	< 0.001
WAISTLINE(cm)	81.61 ± 9.11	93.03 ± 8.81	< 0.001
SBP(mmHg)	111.43 ± 13.86	130.28 ± 16.89	< 0.001
DBP(mmHg)	70.76 ± 9.69	81.92 ± 12	< 0.001
HBA1C(%)	5.57 ± 0.51	6.28 ± 1.21	< 0.001
FASTING_GLUCOSE	91.56 ± 11.69	111.7 ± 31.5	< 0.001
Total cholesterol	190.68 ± 33.28	199.02 ± 40.62	0.036
TG	93.39 ± 54.47	211.32 ± 151.67	< 0.001
HDL_C	55.47 ± 13.8	42.23 ± 9.95	< 0.001
LDL_C	120.61 ± 31.01	122.8 ± 38.01	0.553
BUN	11.98 ± 3.29	13.68 ± 3.87	< 0.001
CREATININE	0.73 ± 0.19	0.81 ± 0.28	0.005
URIC_ACID	5.43 ± 1.39	6.43 ± 1.52	< 0.001
SEX(female)	231(45%)	49(40%)	0.402
Diabetes(%)	0(0%)	15(12%)	< 0.001

P values are calculated from t-test for continuous variables or from chi-square test for categorical

Variables. SBP, systolic blood pressure; DBP, diastolic blood pressure; HDL_C, high density lipoprotein; LDL_C, low density lipoprotein; BUN, blood urea nitrogen

Table 1 show the metabolic syndrome baseline value.

### Spectrum of metabolic syndrome mutant alleles

We searched all alleles in the reference circadian gene and used chi-square test to find whether heterogenous or homogenous genotype is related to metabolic syndrome. Among the genes searched, we found 186 significant SNPs in circadian gene which is associated with metabolic syndrome. (Table 2). In the 186 SNP alleles, we identified 47 alleles associated with hypertension (Table 3), 27 alleles associated with diabetes mellitus (Table 4), 10 alleles associated with low HDL-C (Table 5) and 46 alleles associated with high TG level (Table 6).

**Table 2:** Significant SNPs and odds ratio

Gene refGene	rsId	HO_CI	HO_pvalues	HE_CI	HE_pvalues
GGTLC2;MIR650	rs4050506	1.72–29.82	0.0006	0.01–0.55	0.0003
GGTLC2;MIR650	rs2904924	1.49–15.72	0.0027	0.01–0.65	0.0012
APOL3	rs132653	1.54–82.85	0.0012	0.01–0.65	0.0012
APOL3	rs132651	1.54–82.85	0.0012	0.01–0.67	0.0012
APOL3	rs4821460	1.5–80.84	0.0012	0.01–0.67	0.0012
GGTLC2;MIR650	rs4822280	1.36–6.72	0.0072	0.01–0.74	0.003
GGTLC2;MIR650	rs455194	1.65–28.64	0.001	0.04–0.62	0.001
HPS4	rs56782074	1.37–9.17	0.0138	0.34–0.92	0.0271
TMEM211	rs61643572	1.07–2.4	0.0282	0.37–0.84	0.0061
TMEM211	rs73879166	0.25–0.67	0.0005	1.49–4.03	0.0005
EMID1	rs2857463	0.07–0.81	0.0265	1.24–15.29	0.0265
POM121L1P	rs6003123	1.18–2.62	0.0069	0.35–0.81	0.0038
GGTLC2	rs12484632	1.24–8	0.0122	0.09–0.74	0.004
POM121L1P	rs3876045	1.12–5.1	0.0303	0.21–0.94	0.0428
MYO18B	rs6004865	0.17–0.75	0.0079	1.14–2.52	0.0114
APOL3	rs132650	1.29–7.17	0.0123	0.11–0.71	0.0039
PVALB	rs34262500	1.39–10.92	0.004	0.09–0.72	0.004
APOL3	rs35041494	1.16–3.96	0.0184	0.12–0.75	0.0057
APOL4	rs132718	1.04–11.27	0.0288	0.09–0.96	0.0288
PRAMENP;VPREB1	rs2330036	1.28–8.29	0.0083	0.1–0.78	0.0089
CSF2RB;LL22NC01-81G9.3	rs3950040	1.14–5.26	0.0329	0.38–0.95	0.0382
MYO18B	rs2269635	1.1–2.44	0.0198	0.4–0.92	0.0254
APOL3;APOL4	rs132665	1.35–7.52	0.0084	0.13–0.74	0.0084
LL22NC03-63E9.3;POM121L1P	rs964465	1.24–8	0.0122	0.13–0.84	0.012
POM121L1P	rs3876046	1.02–2.35	0.0479	0.34–0.82	0.0061
RORA	rs11430762	1.08–3.46	0.0324	0.3–0.96	0.0442
LL22NC03-63E9.3;POM121L1P	rs457560	1.24–8	0.0122	0.13–0.86	0.0173
LINC00895;SEPT5	rs5746814	0.19–0.93	0.0405	1.15–2.53	0.0106
LINC00895;SEPT5	rs8143055	0.19–0.93	0.0405	1.13–2.49	0.0134
NULL	rs62228082	1.21–7.85	0.0119	0.09–0.72	0.004
CACNG2	rs4821508	1.13–3.72	0.0254	0.35–0.84	0.0069
GGTLC2;MIR650	rs5759468	1.14–6.38	0.0296	0.16–0.88	0.0296
APOL2	rs132759	1.26–4.95	0.0103	0.18–0.76	0.0076
CACNG2	rs2013924	1.13–3.72	0.0254	0.38–0.89	0.0153
SCARF2	rs759609	1.07–2.52	0.0283	0.34–0.83	0.0075
CACNG2	rs4821506	1.07–3.9	0.0432	0.4–0.94	0.0325
CACNG2	rs2283981	1.13–3.72	0.0254	0.4–0.91	0.0217
NULL	rs60580698	1.1–3.1	0.0254	0.34–0.97	0.047
CES5AP1	rs5751643	1.14–6.38	0.0296	0.17–0.93	0.0425
GGTLC2;MIR650	rs4820531	1.07–6.04	0.0425	0.17–0.93	0.0425

HO_CI, homozygous confidence interval; HE_CI, heterozygous confidence interval

P values are calculated from chi square test

**Table 3:** Hypertension related SNPs

SNP	OR	lower	upper	refGene
rs132759	1.871	1.095	3.423	APOL2
rs132665	1.893	1.011	3.879	APOL3;APOL4
rs2522291	0.696	0.514	0.945	CECR2
rs4820001	1.366	1.023	1.841	CECR3;CECR2
rs5747068	1.367	1.018	1.857	CECR3;CECR2
rs35305666	1.46	1.064	2.035	DERL3
rs5760061	1.454	1.1	1.939	DERL3
rs5760062	1.488	1.079	2.084	DERL3
rs443678	0.466	0.296	0.74	DGCR8
rs2078973	1.473	1.02	2.176	DUSP18;SLC35E4
rs4822280	1.507	1.031	2.347	GGTLC2;MIR650
rs4822932	1.385	1.008	1.891	LOC100507657;MN1
rs66786460	1.409	1.01	1.95	LOC100507657;MN1
rs9612154	1.337	1.03	1.742	MIR650;MIR5571
rs2070455	1.475	1.071	2.062	MMP11
rs5760012	1.502	1.09	2.101	MMP11
rs7289794	1.475	1.071	2.062	MMP11
rs738789	1.466	1.063	2.053	MMP11
rs738789	1.466	1.063	2.053	MMP11
rs60580698	0.793	0.647	0.97	NULL
rs61408070	1.493	1.083	2.088	NULL
Unknow06495	1.868	1.295	2.699	NULL
rs395446	0.459	0.298	0.71	RANBP1;TRMT2A
rs395446	0.459	0.298	0.71	RANBP1;TRMT2A
rs759609	2.164	1.021	5.329	SCARF2
rs6494635	1.875	1.102	3.421	SMAD3
rs10681786	1.46	1.064	2.035	SMARCB1
rs1573277	1.488	1.079	2.084	SMARCB1
rs1972257	1.493	1.083	2.088	SMARCB1
rs1972257	1.493	1.083	2.088	SMARCB1
rs2070458	1.454	1.1	1.939	SMARCB1
rs2073392	1.488	1.079	2.084	SMARCB1
rs2186370	1.454	1.1	1.939	SMARCB1
rs2267039	1.454	1.1	1.939	SMARCB1
rs34378449	1.493	1.083	2.088	SMARCB1
rs5751740	1.502	1.09	2.101	SMARCB1
rs5751741	1.492	1.085	2.083	SMARCB1
rs5760038	1.479	1.075	2.066	SMARCB1
rs5760046	1.508	1.091	2.117	SMARCB1
rs5760046	1.508	1.091	2.117	SMARCB1
rs5760053	1.434	1.03	2.028	SMARCB1
rs5760057	1.51	1.098	2.109	SMARCB1
rs5996620	1.488	1.079	2.084	SMARCB1
rs9608201	1.454	1.1	1.939	SMARCB1
rs174877	0.486	0.3	0.799	TANGO2
rs61643572	1.616	1.06	2.43	TMEM211
rs73879166	1.616	1.06	2.43	TMEM211

OR, odds ratio; lower, lower confidence interval; upper, upper confidence interval

**Table 4:** Diabetes mellitus related SNPs

SNP	OR	lower	upper	refGene	HO
rs403517	1.441	1.049	2.008	BMS1P20;ZNF280B	G/G
rs405570	1.422	1.045	1.96	BMS1P20;ZNF280B	T/T
rs443678	0.599	0.375	0.975	DGCR8	C/C
rs5749150	1.96	1.252	3.215	DUSP18;SLC35E4	G/G
rs12484632	2.398	1.169	5.798	GGTLC2	G/G
rs455194	2.831	1.226	8.232	GGTLC2;MIR650	G/G
rs9623964	0.704	0.511	0.974	IGLL5	C/C
rs457560	3.511	1.54	10.139	LL22NC03-63E9.3;POM121L1P	C/C
rs964465	3.556	1.539	10.335	LL22NC03-63E9.3;POM121L1P	C/C
rs4822932	1.442	1.045	1.978	LOC100507657;MN1	T/T
rs66786460	1.582	1.133	2.194	LOC100507657;MN1	T/T
rs62228082	3.51	1.569	10.034	NULL	G/G
Unknow06495	1.828	1.258	2.66	NULL	T/T
rs140428	3.729	1.742	9.705	POM121L1P	C/C
rs140428	3.729	1.742	9.705	POM121L1P	C/C
rs3876045	2.9	1.397	7.413	POM121L1P	C/C
rs3876046	3.596	1.597	10.313	POM121L1P	G/G
rs6003123	3.424	1.48	9.959	POM121L1P	G/G
rs2330036	0.33	0.121	0.941	PRAMENP;VPREB1	T/T
rs6003527	1.89	1.128	3.355	RAB36	A/A
rs395446	0.6	0.386	0.949	RANBP1;TRMT2A	C/C
rs395446	0.6	0.386	0.949	RANBP1;TRMT2A	C/C
rs61643572	1.681	1.098	2.539	TMEM211	G/G
rs73879166	1.681	1.098	2.539	TMEM211	A/A
rs5993853	2.446	1.183	5.941	TXNRD2	C/C
rs142445063	1.378	1.014	1.898	ZNF280B	A/A
rs2051488	1.369	1.008	1.886	ZNF280B	T/T

OR, odds ratio; lower, lower confidence interval; upper, upper confidence interval

**Table 5:** Low HDL-C related SNPs

SNP	OR	lower	upper	refGene	HO
rs132651	5.443	1.664	33.543	APOL3	C/C
rs132653	5.522	1.671	34.152	APOL3	T/T
rs4821460	5.382	1.627	33.302	APOL3	G/G
rs132718	5.382	1.627	33.302	APOL4	G/G
rs2522291	0.716	0.522	0.988	CECR2	C/C
rs133119	0.643	0.451	0.927	CRYBB2;IGLL3P	C/C
rs635361	1.644	1.038	2.722	CRYBB2P1;GRK3	G/G
rs35305666	1.461	1.045	2.078	DERL3	C/C
rs5760062	1.448	1.033	2.066	DERL3	G/G
rs28411685	2.038	1.255	3.513	DGCR6L;LOC101927859	A/A
rs6518604	1.803	1.141	3.007	DGCR6L;LOC101927859	A/A
rs901790	2.036	1.25	3.516	DGCR6L;LOC101927859	T/T
rs443678	0.443	0.278	0.715	DGCR8	C/C
rs42928	0.676	0.484	0.948	GAL3ST1	T/T
rs4050506	2.151	1.024	5.533	GGTLC2;MIR650	T/T
rs4822280	1.815	1.164	3.14	GGTLC2;MIR650	A/A
rs1005558	0.701	0.531	0.924	ISX;LINC01399	A/A
rs457560	2.564	1.187	6.707	LL22NC03-63E9.3;POM121L1P	C/C
rs964465	2.576	1.174	6.801	LL22NC03-63E9.3;POM121L1P	C/C
rs9617876	2.132	1.265	3.798	LOC101927859	T/T
rs9617876	2.132	1.265	3.798	LOC101927859	T/T
rs5760012	1.417	1.013	2.014	MMP11	A/A
rs33910051	1.493	1.041	2.22	NULL	CCT/CCT
rs61408070	1.452	1.037	2.07	NULL	AC/AC
rs62228082	2.591	1.227	6.691	NULL	G/G
rs28437864	1.578	1.102	2.307	POM121L1P	T/T
rs3876045	1.934	1.013	4.3	POM121L1P	C/C
rs3876046	2.644	1.243	6.858	POM121L1P	G/G
rs6003123	2.48	1.128	6.552	POM121L1P	G/G
rs395446	0.506	0.325	0.799	RANBP1;TRMT2A	C/C
rs395446	0.506	0.325	0.799	RANBP1;TRMT2A	C/C
rs10681786	1.461	1.045	2.078	SMARCB1	ATATCT/ATATCT
rs1573277	1.448	1.033	2.066	SMARCB1	C/C
rs2073392	1.448	1.033	2.066	SMARCB1	G/G
rs34378449	1.452	1.037	2.07	SMARCB1	G/G
rs5751740	1.417	1.013	2.014	SMARCB1	A/A
rs5751741	1.452	1.039	2.066	SMARCB1	A/A
rs5760038	1.44	1.03	2.049	SMARCB1	C/C
rs5760046	1.473	1.048	2.108	SMARCB1	A/A
rs5760046	1.473	1.048	2.108	SMARCB1	A/A
rs5760057	1.469	1.051	2.091	SMARCB1	C/C
rs5996620	1.448	1.033	2.066	SMARCB1	G/G
rs3827341	0.647	0.484	0.864	SYN3	T/T
rs174877	0.387	0.238	0.641	TANGO2	C/C

OR, odds ratio; lower, lower confidence interval; upper, upper confidence interval

**Table 6:** Triglyceride level related SNPs

SNP	OR	lower	upper	refGene	HO
rs132759	2.046	1.227	3.621	APOL2	C/C
rs2283809	0.68	0.51	0.909	CRYBB3	T/T
rs2097195	1.999	1.411	2.89	GGTLC2;MIR650	C/C
rs4822932	1.426	1.056	1.919	LOC100507657;MN1	T/T
rs66786460	1.408	1.026	1.921	LOC100507657;MN1	T/T
rs6004865	0.647	0.455	0.904	MYO18B	C/C
rs200852194	1.497	1.018	2.262	NULL	G/G
rs139726	1.557	1.198	2.035	SGSM1	A/A
rs139728	1.489	1.152	1.935	SGSM1	G/G
rs174877	0.604	0.376	0.983	TANGO2	C/C

OR, odds ratio; lower, lower confidence interval; upper, upper confidence interval

### Gene based prediction model

We applied different machine learning models including logistic regression, random forest, adaboost and neural network to predict metabolic syndrome which is based on gene data. Using our four predicting models (logistic regression, random forest, adaboost and neural network), AUC were 0.68, 0.8, 0.82, 0.8, respectively. The F1 score were 0.424, 0.525, 0.528, 0.526 respectively (for details see Table 7). We chose 40 most significant SNPs in random forest model and used them as the new variable. We compared the 40 most significant OR value with the 40 most important SNPs in random forest model. We found that there are only 11 SNPs overlapping (Table 8) The SNP selected models ((logistic regression, adaboost and neural network) AUC were 0.82, 0.81, 0.85 respectively. The F1 score were 0.578, 0.415, 0.5, respectively (Table 9). Feature selecting models had better performance than original models. The AUC and F1 value are better than previous model.

**Table 7:** Prediction model using all significant SNPs

	AUC	Sens	Spec	Prec	F1
logistic	0.68	0.74	0.586	0.297	0.424
random forest	0.8	0.675	0.788	0.43	0.525
adaboost	0.82	0.764	0.732	0.403	0.528
Neural network	0.8	0.748	0.74	0.405	0.526

AUC, area under curve; Sens, sensitivity; Spec, specificity; Prec, precision value; F1, F1 score

**Table 8:** 40 most important SNPs in random forest model and OR value

RF_SNP	OR_SNP
rs4006261	rs4050506
rs60580698	rs2904924
rs9612154	rs132653
rs66786460	rs132651
rs9605406	rs4821460
rs56782074	rs4822280
rs11430762	rs455194
rs174877	rs56782074
rs2857463	rs61643572
rs133122	rs73879166
rs2283809	rs2857463
rs2331158	rs6003123
rs35251008	rs12484632
rs9606328	rs3876045
rs469995	rs6004865
rs34262500	rs132650
rs6003230	rs34262500
rs377976	rs35041494
rs61643572	rs132718
rs3950040	rs2330036
rs5756977	rs3950040
Unknow06495	rs2269635
rs5998659	rs132665
rs73879166	rs964465
rs131837	rs3876046
rs2254747	rs11430762
rs5748561	rs457560
rs2330036	rs5746814
rs4822689	rs8143055
rs1153417	rs62228082
rs2097195	rs4821508
rs2269635	rs5759468
rs2522291	rs132759
rs17209532	rs2013924
rs9944250	rs759609
rs737855	rs4821506
rs5746814	rs2283981
rs28437864	rs60580698
rs1059142	rs5751643
rs4822932	rs4820531

RF_SNP, Random forest model 40 most important SNP; OR_SNP, 40 most important SNPs according to odds ratio value

**Table 9:** Prediction model using feature selecting SNPs

	AUC	Sens	Spec	Prec	F1
Feature selection	randomforest 40 most important SNPs				
logistic	0.82	0.634	0.89	0.578	0.605
adaboost	0.81	0.772	0.742	0.415	0.54
Neural network	0.85	0.699	0.834	0.5	0.583

AUC, area under curve; Sens, sensitivity; Spec, specificity; Prec, precision value; F1, F1 score

## Discussion

In this study, we found 186 circadian gene SNPs related to metabolic syndrome. Of that there were 8 SNPs related to apolipoprotein. Previous studies have shown that apolipoprotein E knocked out mice will be more likely to developed cardiovascular disease after circadian rhythm was interrupted [31, 32]. Circadian rhythm disorders can alter our body’s metabolic factors including cholesterol profile and apolipoprotein [33]. Another animal study also found that apolipoprotein-E knocked out mice could develop cardiac vascular disease more rapidly after circadian rhythm alteration [34]. Our study also showed that apolipoprotein is related to high TG level, low HDL level and HTN. Rs132759 in APOL2 is both correlated with HTN and low HDL level. Previous studies have shown that APOL2 may be related to acute inflammation response and lipid metabolic processes [35, 36]. To our knowledge, our study is the first to identify that APOL2 is correlated to HTN.

There are 5 SNPs located at BMS1P20 which are long non-coding RNAs (lnc RNA). Previous studies have shown that BMS1P20 is positively corelated to cancer patients’ overall survival especially lung adenocarcinoma [37]. There is also a hypothesis where lnc-RNA regulates our cell by lncRNA-miRNA-mRNA ceRNA network [38]. There are some lnc-RNA reported to be in correlation with metabolism like 116HG, H19, HOTAIR and MIAT [39–41]. We have found rs403517 and rs405570 in BMS1P20 is related to DM, and we believe our study is the first to report BMS1P20 lnc-RNA is related to metabolic syndrome.

MYO18B gene expresses myosin heavy chain that is expressed in human cardiac and skeletal muscle [42]. Some studies showed that MYO18B mutation is associated with myopathy or cardiomyopathy diseases in animal model or in humans [43, 44]. One animal study also show that MYO18B gene expression is regulated by circadian rhythm [45]. In our study, we find that MYO18B is also associated with metabolic syndrome especially rs6004865 which is associated with low HDL levels. Although the SNPs which we find in MYO18B are all intronic or intergenic, we still need more studies to find the relationship between MYO18B and metabolic syndrome.

There are many studies exploring the RORA gene and its relation to circadian rhythm, associated with many psychiatry disorders including major depressive disorder, bipolar disorder, or sleep disturbance disorder [46–48]. RORA gene mutations also affect substance use like alcohol, tea, tobacco or caffeine [47]. This is on a background of the widely accepted knowledge that smoking and alcohol.

consumption will increase the risk of developing metabolic syndrome. The result of an animal system study sees that suppression of RORA gene activity improves metabolic functions and reduces inflammation [49].

Many studies have found that SMARCB1 is a tumor suppressor gene and related to different types of cancer [50]. Recent studies have shown that the circadian clock oscillation was developed during cell differentiation and some cancer cells lack the circadian gene which given the similarity between embryonic stem cell and cancer cell types [51]. Our study found that multiple SNPs in SMARCB1 gene (rs5751740, rs5751741, rs5760038, rs5760046, rs5760057, rs5996620) are both related to high TG level and hypertension. However, the definite mechanism is still unknown.

ZNF280B is an oncogene in the prostate cancer and gastric cancer [52]. Our study is the first to point out that ZNF280B mutation is related to metabolic syndrome. Rs142445063 and rs2051488 are related with diabetes mellitus in our study.

A previous study has used different machine learning method to predict metabolic syndrome. Both clinical information and genetic information were included in the model [53]. In our study, entire dataset or selected SNPs were chosen in different models. The accuracy, AUC value and F1 value were improved in SNPs selected model. Previous studies have showed that feature selection model will have a better performance [54].

The advantage of this study is as follows. First, we examined multiple circadian genes and found multiple SNPs associated with metabolic syndrome. Some SNPs were first found related to metabolic syndrome. Among the significant SNPs, we did subgroup analysis to find out which SNPs corresponds to different metabolic syndrome criteria. Second, based on genetic information; we used four machine learning model to predict metabolic syndrome which to our knowledge has never been performed in previous studies and the AUC value can achieve 0.85 in SNPs selected model.

Nevertheless, there are several limitations in our study. First, the sample size is small and only includes healthy and aware Taiwanese participants. Therefore, this study should be replicated and validated in other populations. Second, this was a cross sectional study. It is difficult for us to find out causal relationships in this study. Third, we only used circadian gene SNPs in our prediction model. Other metabolic syndrome related SNPs or biomarkers can be included to increase accuracy.

## Conclusion

We identified 186 circadian gene SNPs which were related to metabolic syndrome. Among these SNPs, there are 47 alleles associated with hypertension, 46 alleles associated with high serum TG levels, 27 alleles associated with diabetes mellitus and 10 alleles associated with low serum HDL levels. Some SNPs are first found to related with metabolic syndrome. Additional research is needed to confirm these SNPs. In addition, we applied several machine learning models to predict metabolic syndrome based on circadian gene data. We found that it is difficult to produce a high sensitivity model. Other clinical data should be added in to create a higher sensitivity model (Additional files 1, 2, 3, 4, 5, 6, 7, 8).

## Supplementary Information

**Additional file 1: Table S1.** Summary of the 186 significant circadian gene SNPs.

**Additional file 2: Supplementary figure S2** AUC curve of neural network

**Additional file 3: Supplementary figure S3** Precision-Recall curve ofneural network

**Additional file 4: Supplementary figure S4** AUC curve of Adaboost model

**Additional file 5: Supplementary figure S5** Precision-Recall curve of Adaboost model

**Additional file 6: Supplementary figure S6** AUC curve of logisticregression

**Additional file 7: Supplementary figure S7** Precision-Recall curve of logistic regression

**Additional file 8: Supplementary figure S8** Biological pathways-based analysis of circadian rhythm(1)<br>Reference<br>1. Reactome

## Data Availability

The datasets generated and analyzed during the current study are not publicly available due to the privacy regulation of Taiwan biobank but are available from the corresponding author on reasonable request with permission of Taiwan biobank.

## Abbreviations

SNP: Single Nucleotide Polymorphism
AUC: Area under the receiver operating characteristics curve
Mets: Metabolic syndrome
CKD: Chronic Kidney Disease
BMI: Body mass index
SBP: Systolic blood pressure
IDF: The International Diabetes Federation
NAHSIT: Nutrition and Health Survey in Taiwan
TWB: Taiwan Biobank
WGS: Whole genome sequence
HDL: High-density lipoprotein
TG: Triglyceride
