# Supplementary figures and images for: Building a model for predicting metabolic syndrome using artificial intelligence based on an investigation of whole-genome sequencing
Source: J Transl Med. 2022 Apr 28;20:190. doi: 10.1186/s12967-022-03379-7 (PMC9052619; doi:10.1186/s12967-022-03379-7)

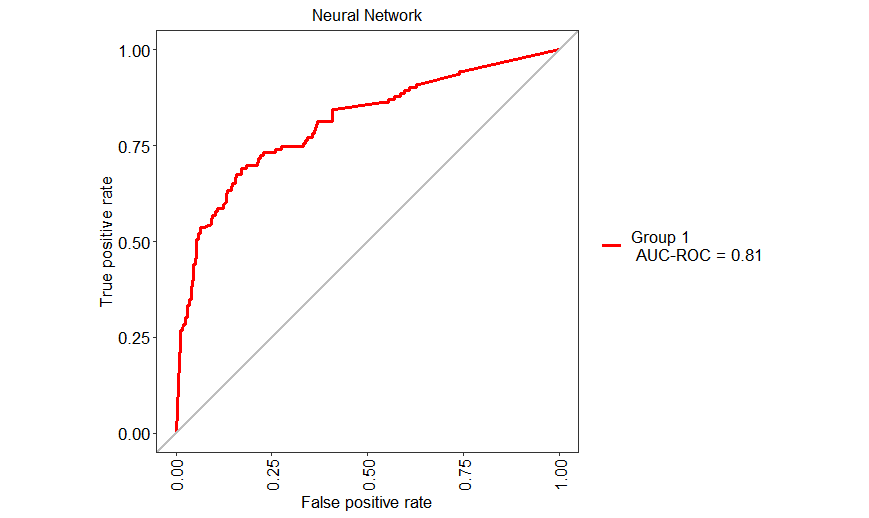


Supplementary figure S2. AUC curve of neural network

Supplement: Supplementary file 2 — Additional file 2: Supplementary figure S2 AUC curve of neural network [file 12967_2022_3379_MOESM2_ESM.docx]

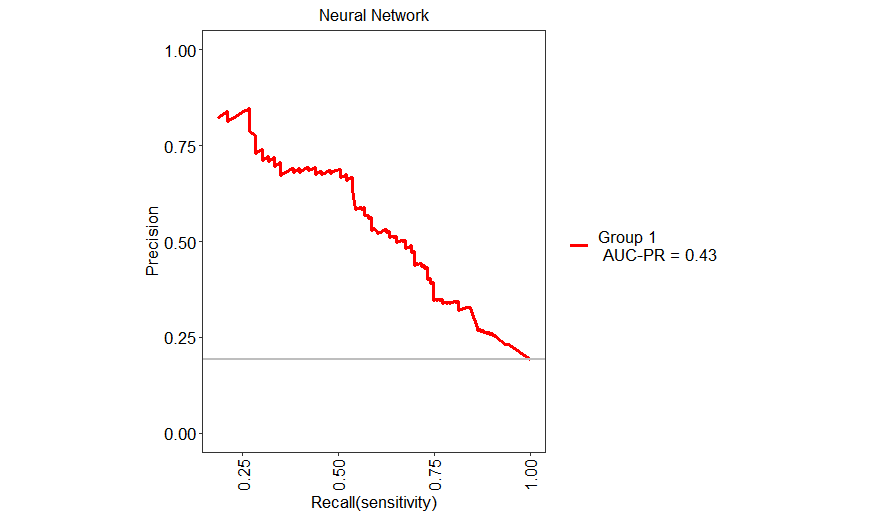


Supplementary figure S3. PR curve of neural network

Supplement: Supplementary file 3 — Additional file 3: Supplementary figure S3 Precision-Recall curve ofneural network [file 12967_2022_3379_MOESM3_ESM.docx]

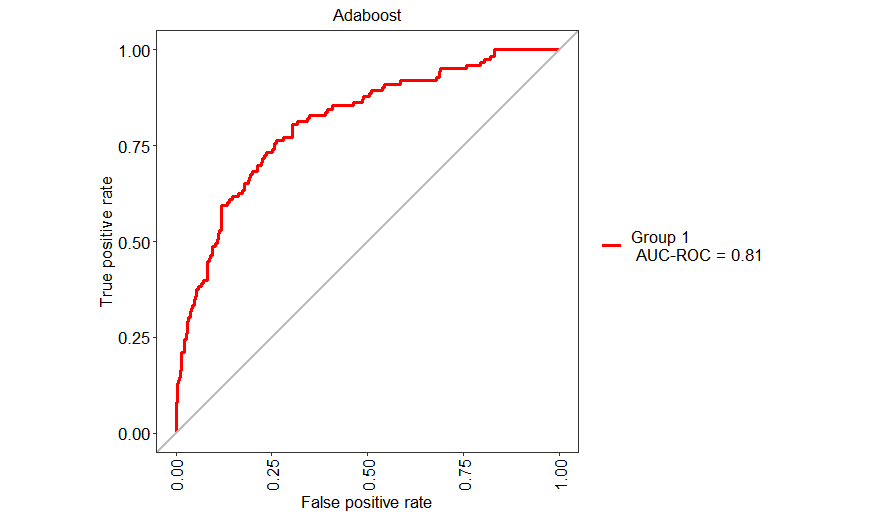


Supplementary figure S4. AUC curve of Adaboost model

Supplement: Supplementary file 4 — Additional file 4: Supplementary figure S4 AUC curve of Adaboost model [file 12967_2022_3379_MOESM4_ESM.docx]

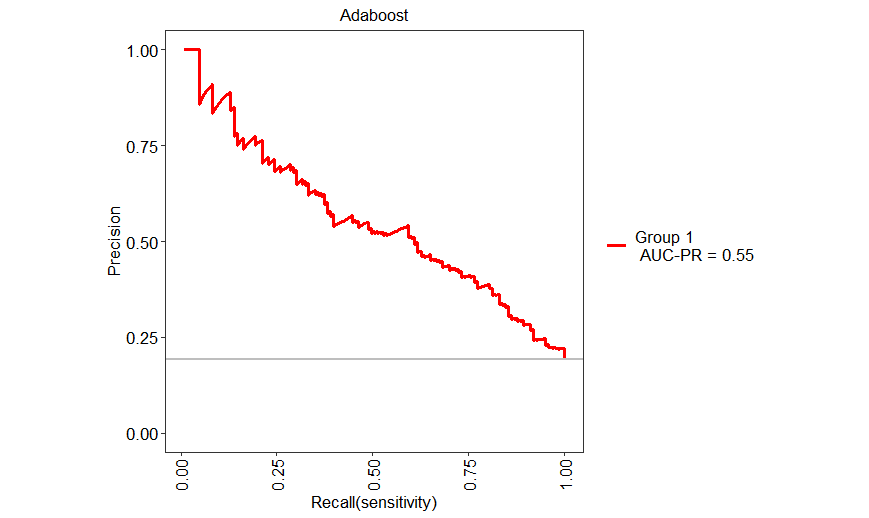


Supplementary figure S5. PR curve of adaboost model

Supplement: Supplementary file 5 — Additional file 5: Supplementary figure S5 Precision-Recall curve of Adaboost model [file 12967_2022_3379_MOESM5_ESM.docx]

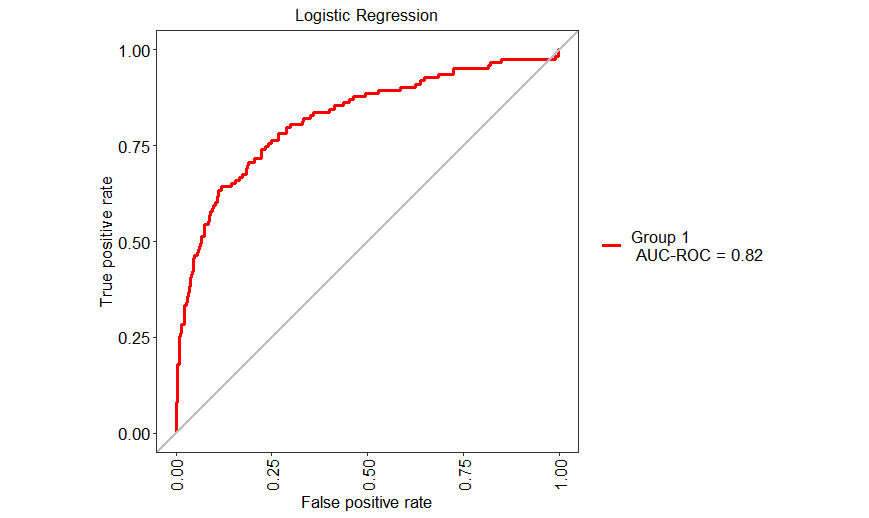


Supplementary figure S6. AUC curve of logistic regression

Supplement: Supplementary file 6 — Additional file 6: Supplementary figure S6 AUC curve of logisticregression [file 12967_2022_3379_MOESM6_ESM.docx]

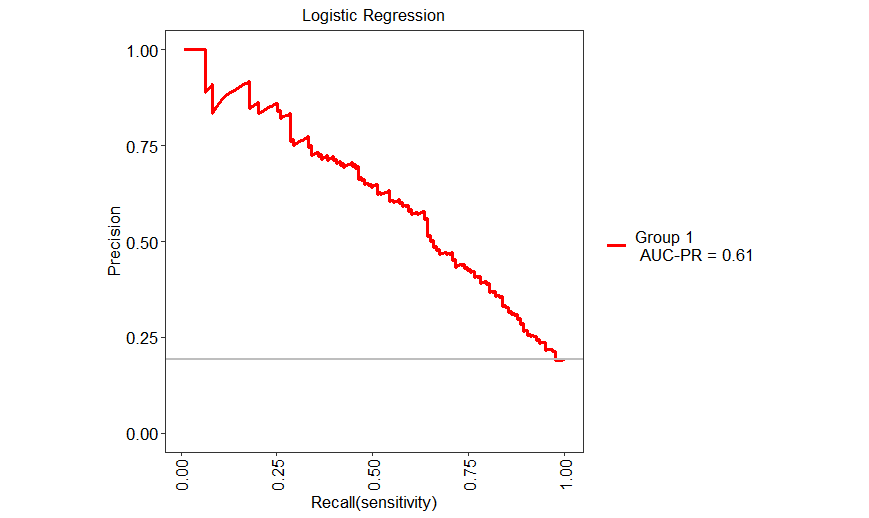


Supplementary figure S7. PR curve of logistic regression

Supplement: Supplementary file 7 — Additional file 7: Supplementary figure S7 Precision-Recall curve of logistic regression [file 12967_2022_3379_MOESM7_ESM.docx]
