# Supplementary material for: Building a model for predicting metabolic syndrome using artificial intelligence based on an investigation of whole-genome sequencing
Source: J Transl Med. 2022 Apr 28;20:190. doi: 10.1186/s12967-022-03379-7 (PMC9052619; doi:10.1186/s12967-022-03379-7)
Supplement: Supplementary file 8 — Additional file 8: Supplementary figure S8 Biological pathways-based analysis of circadian rhythm(1)Reference1. Reactome [file 12967_2022_3379_MOESM8_ESM.docx]

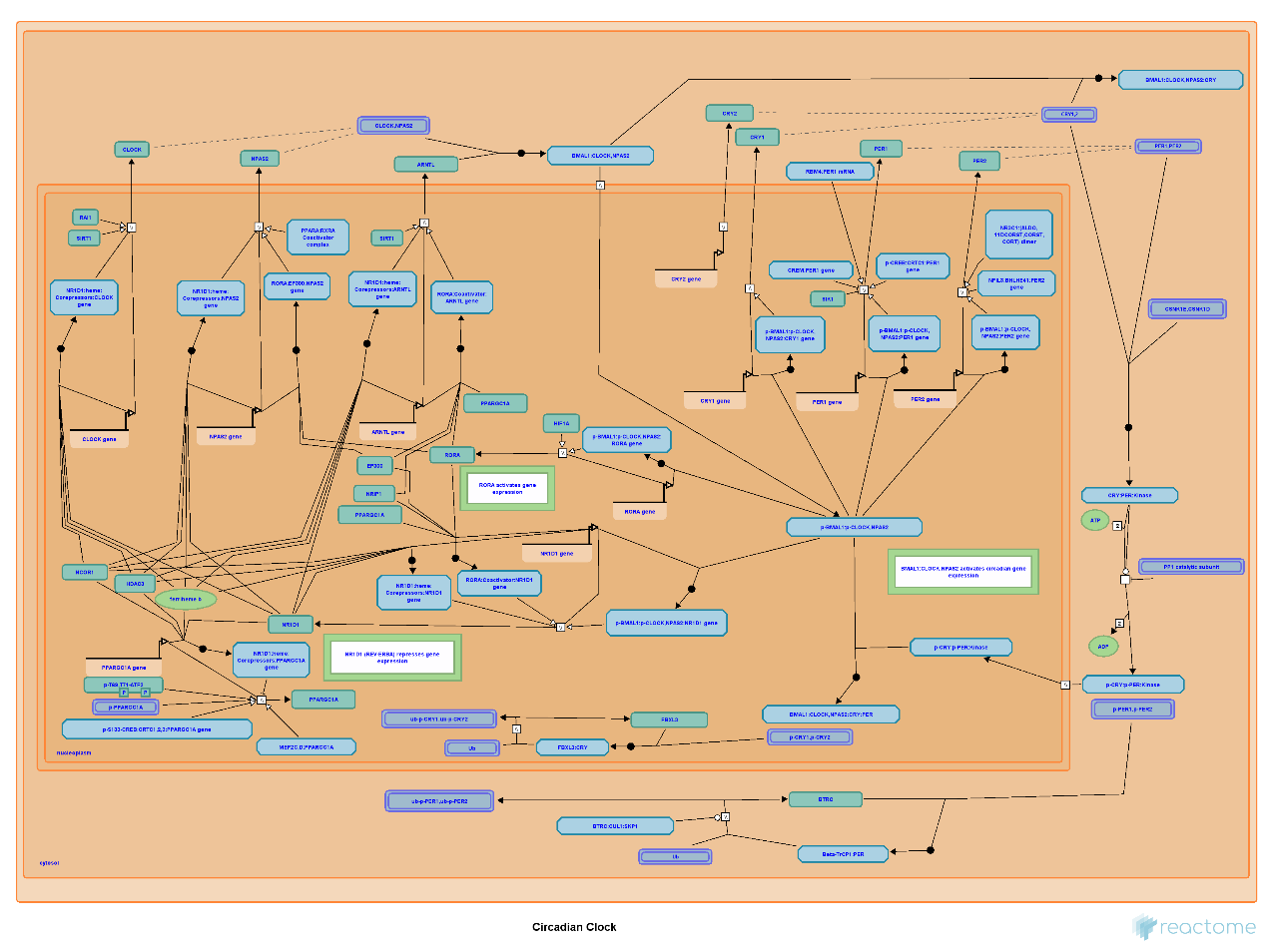


Supplementary figure S8. Biological pathways-based analysis of circadian rhythm(*1*)

Reference

1. Reactome.
